# Effects of *Taraxacum mongolicum Hand.-Mazz.* (dandelion) on growth performance, expression of genes coding for tight junction protein and mucin, microbiota composition and short chain fatty acids in ileum of broiler chickens

**DOI:** 10.1186/s12917-022-03278-5

**Published:** 2022-05-14

**Authors:** Jinju Mao, Yuan Wang, Wenwen Wang, Ting Duan, Na Yin, Tao Guo, Hui Guo, Na Liu, Xiaoping An, Jingwei Qi

**Affiliations:** 1 College of Animal Science, Inner Mongolia Agricultural University, Hohhot, 010018 China; 2 Inner Mongolia Herbivorous Livestock Feed Engineering Technology Research Center, Hohhot, 010018 China; 3 Key Laboratory of Smart Animal Husbandry at Universities of Inner Mongolia Automomous Region, Hohhot, 010018 China

**Keywords:** Dandelion, Growth performance, Barrier function, Microbiota composition, Broiler

## Abstract

**Background:**

Dandelion is becoming an exploitable alternative to the widely prohibited antibiotics in the poultry production. This research aimed to investigate the effects of dandelion on the growth performance and intestinal barrier function of broiler chickens maintained under standard condition of management. One-hundred and sixty 1-day-old Arbor Acres (AA) male broiler chickens were randomly divided into four groups, with five replicates of eight birds each. The birds were fed a basal diet supplemented without (control group, [CON]) or with 500 (low dose [LD]) or 1000 (high dose [HD]) mg/kg dandelion or with 250 mg/kg chlortetracycline 20% premix (CTC) for 42 days, including the starter phase (d 1 to 21) and the grower phase (d 22 to 42). Body weight (BW) of each bird and feed consumption of each replicate were measured at d 21 and d 42. The ileal tissues were collected on day 21 and 42 to determine expression of genes coding for tight junction protein and mucin as well as ELISA analysis for immune factor. The ileal digesta was collected for microbiota and short chain fatty acids analysis.

**Results:**

Compared with CON group, during day 1–21, the average daily feed intake (ADFI) and feed/gain ratio (F/G) of LD group were lower (*P* < 0.05); during day 22–42, the F/G of LD and CTC group tended to be lower (*P* = 0.07); during the overall phase, the ADFI of HD and CTC groups were decreased (*P* < 0.05), and the F/G of dandelion and CTC groups tended to be decreased (*P* = 0.07). On day 21, the relative mRNA expression of claudin, occludin-1 and mucin1 in dandelion groups were up-regulated (*P* < 0.05), and the ZO-1 mRNA expression in CTC group was increased (*P* < 0.05); on day 42, the claudin and mucin1 transcripts in LD group and ZO-1 transcripts in HD and CTC group were up-regulated (*P* < 0.05), while the occludin-1 and mucin1 transcripts in CTC group was significantly down-regulated (*P* < 0.05). In addition, the contents of TNF-α in dandelion groups were lower than that in CTC group (*P* < 0.05). In the analysis of ileal microbiota, on day 21, decreased α-diversity was observed in HD and CTC groups (*P* < 0.05). Meanwhile, on day 21, the relative abundance of Firmicutes in dandelion groups tended to be higher (*P* = 0.09), the relative abundance of *Lactobacillus* in LD and CTC group were increased (*P* < 0.05), while Bacteroidete, *Bacteroides*, and *Alistipes* relative abundance in dandelion and CTC groups were decreased (*P* < 0.05). On day 42, the Actinobacteriota relative abundance in CTC group tended to be higher (*P* = 0.05), and *Lysinibacillus* relative abundance of CTC group was higher (*P* = 0.02). Compared with CON group, on day 21, the propionic acid and butyric acid content in CTC group were higher, the butyric acid content in HD group was lower (*P* < 0.10).

**Conclusion:**

In summary, dietary dandelion supplementation at 500 mg/kg of diet enhanced growth performance of broilers by improving the intestinal barrier function. Dandelion can be supplemented in the diet as an antibiotics alternative to enhance production in poultry industry.

## Background

The complete intestinal barrier composed of physical, chemical, immunological, and microbiological barriers plays a pivotal role in preventing invasion of pathogens and toxins from the intestines [1, 2]. It is well known that the damage of intestinal barrier is closely related to the intestinal diseases and growth inhibition of broilers as well as the economic loss of poultry production. Hence, the intactness of intestinal barrier is crucial for the wellness and productivity of broilers. Previously, antibiotics have been widely used as feed additives in order to prevent diseases and promote growth of broilers [3, 4]. However, the abuse of antibiotics has resulted in serious consequences, such as antimicrobial resistance and antibiotic residues, all of which represent serious threats to the health of both animals and human [5–7]. From July 1, 2020, China has completely banned the usage of antibiotics as feed additives in animal husbandry according to the Ministry of Agriculture and Rural Affairs Announcement No. 194. Therefore, the identification of feed additives that can improve the intestinal barrier function and growth performance is an effective strategy for alternating antibiotics for poultry production.

Among the alternatives, Chinese herbs have received a lot of attention due to their modulating effects on nutritional metabolism, immune response, and intestinal health of monogastric animals [8]. Dandelion (*Taraxacum mongolicum* Hand.-Mazz.) is a kind of Chinese herbs (CH) with a long history in China [9]. Dandelion contains variety of bioactive substances, such as polysaccharides, polyphenols and terpenoids [10], and has been proved to display anti-inflammatory and antibacterial activities in vitro [11, 12]. Like other CH, the growth promoting effects of dandelion has been demonstrated in study with pigs [13]. Additionally, researches on golden pompano (*Trachinotus ovatus*) have shown that dietary dandelion extracts supplementation enhanced the growth performance by modulating the richness and diversity of gut microbiota and regulating the mRNA expression levels of tight junction proteins and immune-related genes [14, 15]. However, no data is available regarding the effects of dandelion on growth performance and intestinal barrier functions of broilers. Therefore, we hypothesized that dietary supplementation with dandelion could enhance the growth performance of broilers by improving intestinal barrier function. Thus, the aim of this study was to investigate the effects of dandelion on growth performance, expression of genes coding for tight junction protein and mucin, microbiota composition and SCFA in ileum of broiler chickens.

## Results

### Growth performance

The effects of dandelion on growth performance of broilers are shown in Table 1. There was no significant difference in the initial weight of birds among four groups (*P* > 0.05). Throughout the experimental periods, supplementation of dandelion and CTC did not affect the BW and ADG of broilers compared with that of the CON group (*P* > 0.05). During day 1–21, birds in LD group had significantly lower ADFI and F/G (*P* < 0.05) than those in CON group. Further, the F/G of LD and CTC group tended to be lower (*P* = 0.07) than that of CON group during day 22–42. During the whole period, compared to that in the CON group, the ADFI in HD and CTC group was significantly decreased (*P* = 0.02). In addition, the F/G of dandelion and CTC groups tended to be decreased (*P* = 0.07).

**Table 1:** Effects of dandelion on growth performance of broilers

Items	Treatment group				SEM	*P-Value*
	CON	LD	HD	CTC		
BW						
1 day	44.48	44.34	44.67	45.42	0.321	0.68
21 day	718.86	785.62	768.72	739.14	11.688	0.18
42 day	2319.80	2569.90	2425.90	2355.30	40.795	0.13
1 to 21 day						
ADG/g	31.86	35.75	34.56	33.22	0.602	0.11
ADFI/g	47.84^a^	40.18^b^	44.42^ab^	46.90^a^	1.002	0.01
F/G	1.48^a^	1.14^b^	1.34^a^	1.43^a^	0.041	< 0.01
22 to 42 day						
ADG/g	76.06	84.65	79.05	78.24	1.573	0.27
ADFI/g	139.79	145.38	130.81	129.33	2.770	0.12
F/G	1.86^x^	1.70^y^	1.71^xy^	1.67^y^	0.028	0.07
1 to 42 day						
ADG/g	54.19	60.12	56.62	55.49	0.987	0.17
ADFI/g	93.81^a^	93.77^a^	86.45^b^	85.59^b^	1.362	0.02
F/G	1.74^x^	1.56^y^	1.57^y^	1.58^y^	0.030	0.07

*CON* The control treatment, *LD* The low dose of dandelion treatment, *HD* The high dose of dandelion treatment, *CTC* The chlortetracycline treatment, *SEM* Standard error of the mean

^a,b^Means (*n* = 5) in a row with different letters differed significantly (*P* < 0.05)

^x,^^y^Means (*n* = 5) in a row with different letters tended to be different (0.05 ≤ *P* < 0.10)

### Gene expression of tight junction proteins and mucin

The effects of dandelion on the gene expression of tight junction proteins and mucin in ileum tissue of broilers are showed in Table 2. In comparison with CON group, the relative mRNA expression of claudin, occludin-1 and mucin1 in dandelion group was significantly up-regulated (*P* < 0.05) on day 21, and birds in CTC group had significantly increased relative mRNA expression of ZO-1 (*P* < 0.05). On day 42, relative mRNA expression of claudin, occludin-1, and mucin1 in LD group was significantly higher than that of CON group (*P* < 0.05). Meanwhile, the relative mRNA expression of ZO-1 of HD and CTC group was significantly increased (*P* < 0.05). However, the relative mRNA expression of occludin-1 and mucin1 in CTC group was significantly down-regulated compared with CON group (*P* < 0.05).

**Table 2:** Effects of dandelion on ileal expression of genes coding for tight junction protein and mucin of broilers

Items	Treatment group				SEM	*P*-Value
	CON	LD	HD	CTC		
21 day						
Claudin	1.01^c^	2.28^b^	3.85^a^	0.64^c^	0.318	< 0.01
Occludin-1	1.01^c^	2.19^b^	3.63^a^	0.70^c^	0.295	< 0.01
ZO-1	0.93^bc^	0.69^c^	1.48^b^	2.00^a^	0.149	< 0.01
Mucin1	0.92^b^	1.88^a^	2.31^a^	1.03^b^	0.161	< 0.01
42 day						
Claudin	1.01^b^	2.31^a^	1.25^b^	0.93^b^	0.151	< 0.01
Occludin-1	1.00^b^	1.38^a^	0.76^bc^	0.62^c^	0.085	< 0.01
ZO-1	1.06^b^	1.22^b^	2.72^a^	2.64^a^	0.190	< 0.01
Mucin1	1.01^b^	1.89^a^	0.84^bc^	0.68^c^	0.121	< 0.01

*CON* The control treatment, *LD* The low dose of dandelion treatment, *HD* The high dose of dandelion treatment, *CTC* The chlortetracycline treatment, *SEM* Standard error of the mean

^a,b,c^Means (*n* = 5) within a row with different letters differed significantly (*P* < 0.05)

### Ileal immune factor

Table 3 shows the effects of dandelion on the ileal immune factor in ileum tissue of broilers. The content of TNF-α in the dandelion groups was lower than that in CTC group (*P* < 0.05), while ﻿similar to that of the CON group.

**Table 3:** Effects of dandelion on ileal immunity of broilers. (ng/g protein)

Items	Treatment group				SEM	*P-Value*
	CON	LD	HD	CTC		
21 day						
sIgA	379.43	405.13	424.38	428.07	18.889	0.80
IL-1	40.02	36.98	39.74	42.60	1.465	0.67
IL-2	96.85	85.01	77.17	82.13	4.925	0.55
IL-10	31.25	43.83	41.18	43.47	2.720	0.36
TNF-α	44.86^ab^	35.81^b^	34.75^b^	49.25^a^	2.219	0.04
42 day						
sIgA	381.33	371.96	442.15	476.59	18.078	0.11
IL-1	37.97	35.05	45.21	39.09	2.364	0.52
IL-2	66.75	73.81	80.64	66.35	4.254	0.63
IL-10	37.71	36.56	37.81	35.81	2.088	0.99
TNF-α	39.50	37.85	38.43	38.07	1.905	0.99

*CON* The control treatment, *LD* The low dose of dandelion treatment, *HD* The high dose of dandelion treatment, *CTC* The chlortetracycline treatment, *SEM* Standard error of the mean

^a,b^Means (*n* = 5) within a row with different letters differed significantly (*P* < 0.05)

### Ileal microbiota

The alpha diversity of ileal microbiota is shown in Table 4. Compared with CON group, on day 21, the observed_species, ace, and chao1 indices of HD and CTC group were significantly lower (*P* < 0.05). The shannon indice in dandelion and CTC groups was significantly decreased (*P* = 0.01). Additionally, birds in dandelion groups had significantly lower simpson indice (*P* = 0.02). On day 42, the simpson indice of HD and CTC group tended to be higher than that of CON group (*P* = 0.06).

**Table 4:** Effects of dandelion on the alpha-diversity of ileal microbiota of broilers

Items	Treatment group				SEM	*P-Value*
	CON	LD	HD	CTC		
21 day						
observed_species	277^a^	178^ab^	136^b^	135^b^	20.226	0.03
shannon	4.05^a^	2.86^b^	2.32^b^	2.80^b^	0.204	0.01
simpson	0.87^a^	0.72^b^	0.62^b^	0.74^ab^	0.031	0.02
ace	280.76^a^	184.36^ab^	142.48^b^	145.15^b^	19.580	0.03
chao1	279.94^a^	180.82^ab^	136.95^b^	137.36^b^	20.244	0.02
42 day						
observed_species	432	334	410	571	37.701	0.16
shannon	2.72	3.14	3.52	4.11	0.240	0.22
simpson	0.58^y^	0.69^xy^	0.80^x^	0.85^x^	0.041	0.06
ace	483.00	387.50	458.50	624.46	39.969	0.20
chao1	475.80	367.40	458.70	624.81	39.534	0.13

*CON* The control treatment, *LD* The low dose of dandelion treatment, *HD* The high dose of dandelion treatment, *CTC* The chlortetracycline treatment, *SEM* Standard error of the mean

^a,b^Means (*n* = 5) within a row with different letters differed significantly (*P* < 0.05)

^x,^^y^Means (*n* = 5) in a row with different letters tended to be different (0.05 ≤ *P* < 0.10)

The effects of dandelion on the relative abundances of ileal microbiota at the phyla level are shown in Table 5. Firmicutes was the dominant phylum in ileal microbiota at 21 and 42 d. Compared with CON group, on day 21, the relative abundance of Firmicutes in dandelion groups tended to be increased (*P* = 0.09), while the relative abundance of Bacteroidete in dandelion and CTC groups was significantly decreased (*P* < 0.01). Further, the relative abundance of Actinobacteriota in CTC group tended to be increased in comparison with CON group on day 42 (*P* = 0.05).

**Table 5:** Effects of dandelion on the relative abundances of ileal microbiota at the phyla level (%)^1^

Items	Treatment group				SEM	*P-Value*
	CON	LD	HD	CTC		
21 day						
Firmicutes	74.26^y^	90.57^x^	92.29^x^	87.57^xy^	2.687	0.09
Cyanobacteria	11.06	6.36	4.86	8.28	1.574	0.57
Proteobacteria	3.25	1.91	2.55	3.49	0.484	0.69
Bacteroidetes	9.01^a^	0.82^b^	0.13^b^	0.22^b^	1.169	< 0.01
42 day						
Firmicutes	86.04	90.62	73.78	81.47	3.352	0.35
Cyanobacteria	4.99	0.83	14.15	10.73	2.471	0.24
Proteobacteria	4.74	6.28	9.54	3.67	1.286	0.42
Bacteroidetes	3.12	1.19	1.60	2.08	0.530	0.64
Actinobacteriota	0.33^y^	0.40^y^	0.35^y^	1.17^x^	0.131	0.05

*CON* The control treatment, *LD* The low dose of dandelion treatment, *HD* The high dose of dandelion treatment, *CTC* The chlortetracycline treatment, *SEM* Standard error of the mean

^1^Only bacteria with a relative abundance greater than 1% are shown in the table

^a,b^Means (*n* = 5) within a row with different letters differed significantly (*P* < 0.05)

^x,^^y^Means (*n* = 5) in a row with different letters tended to be different (0.05 ≤ *P* < 0.10)

Table 6 shows the effects of dandelion on the relative abundances of ileal microbiota at the genera level. As reported in Table 6, *Lactobacillus*, belonging to Firmicutes, was the dominant genus in ileal microbiota. Compared with CON group, on day 21, the relative abundance of *Lactobacillus* in the LD and CTC group was significantly increased (*P* = 0.01), while the relative abundance of *Bacteroides* and *Alistipes* in dandelion and CTC groups were significantly decreased (*P* < 0.05). Additionally, the relative abundance of *Lysinibacillus* of CTC group was significantly higher than that of CON group on day 42 (*P* = 0.02).

**Table 6:** Effects of dandelion on the relative abundances of ileal microbiota at the genera level (%)^1^

Items	Treatment group				SEM	*P-Value*
	CON	LD	HD	CTC		
21 day						
* Lactobacillus*	39.21^b^	64.20^a^	62.68^ab^	80.89^a^	5.071	0.02
* Romboutsia*	5.79	9.05	3.76	9.76	1.793	0.64
* Candidatus Arthromitus*	8.99	8.46	3.50	5.67	1.352	0.47
* unidentified Chloroplast*	11.06	6.36	4.86	8.28	1.574	0.57
* Escherichia Shigella*	0.94	0.66	1.14	1.54	0.270	0.74
* Enterococcus*	2.81	2.57	1.11	2.14	0.499	0.68
* Bacteroides*	5.73^a^	0.67^b^	0.07^b^	0.15^b^	0.783	0.01
* Streptococcus*	2.90	0.93	0.83	1.29	0.626	0.66
* unidentified Mitochondria*	1.87	0.93	0.71	1.41	0.298	0.56
* Alistipes*	3.27^a^	0.15^b^	0.07^b^	0.07^b^	0.451	0.01
42 day						
* Lactobacillus*	80.52	83.70	63.77	61.48	4.053	0.11
* unidentified Chloroplast*	4.97	0.62	14.14	10.71	2.478	0.23
* Escherichia Shigella*	3.28	0.81	6.00	1.03	1.123	0.34
* Pseudomonas*	0.58	1.85	2.13	1.11	0.318	0.31
* Bacteroides*	1.88	0.37	0.42	0.68	0.347	0.40
* Candidatus Arthromitus*	0.46	1.44	0.75	1.28	0.250	0.51
* Lysinibacillus*	0.07^b^	0.10^b^	0.87^ab^	2.61^a^	0.357	0.02

*CON* The control treatment, *LD* The low dose of dandelion treatment, *HD* The high dose of dandelion treatment, *CTC* The chlortetracycline treatment, *SEM* Standard error of the mean

^1^Only bacteria with a relative abundance greater than 1% are shown in the table

^a,b^Means (*n* = 5) within a row with different letters differed significantly (*P* < 0.05)

### Ileal Short chain fatty acids (SCFA) content

The effects of dandelion on the SCFA content in the ileal digesta of broilers are shown in Table 7. The results show that acetic acid is the most abundant SCFA in the ileum in all groups. On day 21, the propionic acid content in HD group tended to be lower than that in CTC group (*P* = 0.07), while did not differ to that of the CON group. Compared with CON group, butyric acid content in HD was significantly decreased, while that in CTC group was significantly increased (*P* = 0.01). No difference in SCFA content of ileal digesta on day 42 was observed among dietary treatments (*P* > 0.05).

**Table 7:** Effects of dandelion on the SCFA concentrations in ileal digesta of broilers. (μg/g)

Items	Treatment group				SEM	*P-Value*
	CON	LD	HD	CTC		
21 day						
Acetic acid	8.03	7.78	6.18	5.04	0.952	0.67
Propionic acid	0.37^xy^	0.40^xy^	0.25^y^	0.99^x^	0.125	0.07
Butyric acid	0.17^b^	0.25^ab^	0.15^c^	0.28^a^	0.018	0.01
Total SCFA	8.41	9.59	7.95	6.31	1.000	0.71
42 day						
Acetic acid	7.23	9.02	4.26	6.66	1.026	0.46
Propionic acid	0.30	0.51	0.12	0.11	0.083	0.26
Butyric acid	0.05	0.09	0.18	0.05	0.036	0.56
Total SCFA	7.52	9.63	4.57	6.82	1.093	0.46

*CON* The control treatment, *LD* The low dose of dandelion treatment, *HD* The high dose of dandelion treatment, *CTC* The chlortetracycline treatment, *SEM* Standard error of the mean

^a,b^Means (*n* = 5) within a row with different letters differed significantly (*P* < 0.05)

^x,^^y^Means (*n* = 5) in a row with different letters tended to be different (0.05 ≤ *P* < 0.10)

## Discussion

Dandelion, belongs to the family Asteraceae, has received widespread attention due to its growth promoting benefits, which is mainly attributed to its bioactive substances, such as polysaccharides, polyphenols and terpenoids [16]. Dietary supplementation with dandelion enhanced the growth performance of pigs [13] and olden pompano *Trachinotus ovatus* [14, 15]. Noor et al. (2021) reported that dietary supplementation of 2.0 g/kg dandelion leaf powder decreased the feed consumption and feed conversion factor of broiler chickens [17]. In consistent with previous studies, the present study showed that adding dandelion to the diet decreased ADFI and F/G ratio of broilers, and that dandelion supplementation at low dosage in particular, improved broiler performance more than high dosage and CTC. This positive effect of the dandelion on the growth performance may be due to the improvement in intestinal barrier functions [14, 15]. However, in this study, growth performance responses to dandelion at low dosage appeared to be better than high dosage. It is believed that effects of active components from herbs and spices depends largely on the dosage used and excess amounts can be even toxic [18]. However, the reason will need to be further studied to be confirmed.

The integrity of intestinal barrier function (physical, chemical, immunological and microbiological barriers) is very important for defense against invasion of harmful substances [1]; thus, impairment of intestinal barrier function could initiate and promote health and growth of broilers. The physical barrier is composed of intestinal mucosal epithelial cells and their tight junctions, including claudins, occludin and zonula occludens (ZO) [19, 20]. Mucins secreted by intestinal goblet cells are important components of the intestinal chemical barrier [21, 22]. In a previous study, it is found that dandelion extracts enhanced the mRNA expression of ZO-1 and occludin of golden pompano [15]. Additionally, dietary supplementation with polysaccharides from *Atractylodes macrocephalae* Koidz., which belongs to the Asteraceae family, enhanced the mRNA and protein expressions of mucin 2 and tight junction protein claudin-1 in the colon of DSS-induced mice [23]. However, to the best of our knowledge, no study has been conducted to examine the effects of dandelion on the intestinal physical and chemical barrier function of broilers. In this study, the addition of dandelion up-regulated the relative mRNA expression of tight junction protein and mucin. These results indicated that dandelion could improve intestinal physical and chemical barrier function of broilers. The improvement of intestinal physical and chemical barrier function may be partly associated with the antibacterial activity of dandelion [16], which modulates intestinal microbiota and their metabolites, and then limits pathogenic bacteria access to the epithelial surface [24, 25].

Another particularly important part of intestinal barrier is the immunological barrier. Secretory IgA antibodies are the most important humoral immune factors present on mucosal surfaces [26]. A recent study showed that total flavonoids from *Eupatorium odoratum* L. (Asteraceae family) increased the sIgA level in duodenal mucosa of broiler chicken [27]. However, in this study, dietary dandelion inclusion failed to induce any effect on the ileal sIgA level of broilers. Cytokines exert an important biological function in immunomodulatory function and can be divided into anti-inflammatory cytokines and pro-inflammatory cytokines. Previous study has pointed out that over-production of pro-inflammatory cytokines (e.g., TNF-α) decreased tight junction proteins protein expression and impair intestinal integrity [28]. It is reported that dandelion inhibits TNF-α production from rat astrocytes stimulated by lipopolysaccharide (LPS) [29]. Similarly, polysaccharides derived from *Echinacea purpurea* (family Asteraceae) protected intestinal epithelial cells from LPS induced injury by decreasing TNF-α mRNA expression [30]. Consistent with previous studies, feeding dandelion to a certain extent reduced the TNF-α concentration in ileal tissue of broilers in this study. This might further support the fact that dandelion could inhibit inflammation response and improve intestinal integrity.

The microbiological barrier is represented by the intestinal microbiota, referring to the entire population of microorganisms colonizing the intestinal tract [31]. Intestinal microbiota and related metabolites have been shown to influence nutritional metabolism, immune response, and barrier function of host [32, 33]. Alpha diversity is always used to analyze the complexity of species diversity [34]. Tan et al. reported beneficial effects of dietary dandelion extract inclusion on alpha diversity of intestinal microbiota in golden pompano [14]. However, it is found that dandelion decreased the alpha diversity of ileal intestinal microbiota of broilers in the current study. The present study provided the most detailed description of intestinal microbiota of broiler response to dandelion to date; thus, it is difficult to make any direct comparison. It is speculated that the decreases in alpha diversity of ileal intestinal microbiota of broilers may be also related to the antibacterial activity [35, 36], because higher relative abundance of core microbiota (Firmicutes and *Lactobacillus*) and lower relative abundance of Bacteroidetes, *Bacteroides* and *Alistipes* were observed in this study. It is suggested that compared to Bacteroidetes, Firmicutes provide more effective energy source for intestine cell, thus consequently promoting carbohydrate absorption and increasing weight gain [37]. *Lactobacillus* species are probiotics, which inhibit the growth of pathogenic bacteria by the production of lactic acid [38]. This tendency was consistent with the result of growth performance, and both indicated the intestinal barrier could be enhanced by dietary dandelion supplementation in the present study. Similar to our results, dietary sanguinarine supplementation increased the relative abundance of Firmicutes, and decreased the relative abundance of Bacteroidetes in the cecal digesta of broiler chickens [37]. In addition, Wu et al. reported that the extract (rich in polyphenol) from *Pandanus tectorius* fruit enhanced the relative abundance of *Lactobacillus* and decreased the relative abundance of *Bacteroides* and *Alistipes* [39]. ﻿

Short-chain fatty acids (SCFAs) are the main metabolic end products of intestinal microbiota, which are not only an important energy source for the gut microbiota itself and intestinal epithelial cells, but also an important regulator in the growth and colonization of pathogens and the expression tight junction proteins [25, 40, 41]. In a preview study, dandelion was found to increase acetic acid and butyric acid contents in rumen of lactating dairy cows [42]. Administration of Achillea L. (Compositae or Asteraceae) showed effectiveness in protecting the gastric mucosa of rats by enhancing acetic acid production [43]. Additionally, inulin, a polysaccharide from root of *Helianthus tuberosus* (Asteraceae family) exerts prebiotic effects because it could be fermented to SCFAs, especially butyric acid, by gut microbiota [44]. Unfortunately, no other studies using dandelion have reported data on the SCFAs content in ileal digesta of broilers. In this study, the contents of total SCFAs, propionic acid and butyric acid in the LD group were increased compared with the CON group, while that of HD group were decreased. The possible explanation of the differences in results received from low and high dosage of dandelion could be that excess dandelion extracts might results in toxic effects and stress in animals [18].

CTC is one of the commonly used antibiotics in animal husbandry. Antibiotics have been acknowledged to improve feed conversion and growth and reduce morbidity and mortality caused by clinical and subclinical diseases. A preview study with germ-free chicken indicates that the growth promoting effects of antibiotics are mediated by their antimicrobial activity, which reduce the microbial load in the gut and increase nutrient availability for the host [45]. In our study, the positive effects of CTC were confirmed by decreases in the ADFI and F/G of broiler chickens and increases the gene expression of ZO-1, the relative abundance of Antinobacterota, *Lactobacillus*, and *Lysinibacillus*, and the content of propionic acid and butyric acid. In addition, our study showed that compared to CTC group, LD group had lower ADFI, F/G and ileal TNF-α content while higher tight junction proteins and mucin gene expressions. These results indicated that dandelion has the potential to replace CTC and good prospects of development and utilization in poultry production.

## Conclusions

In summary, the present study indicated that dandelion administration at 500 mg/kg of diet could enhance the feed utilization of broilers by strengthening intestinal barrier function through enhancing gene expression of tight junction proteins and mucin, decreasing pro-inflammatory cytokines, and improving the structure of intestinal microbiota. Dandelion can be supplemented in the diet as an antibiotics alternative to enhance production in poultry industry.

## Methods

### Ethics statement

The ethics of the experimental protocol were approved by Inner Mongolia Agricultural University Research Ethics Committee (permission number of [2020]065). All animal experiments were performed following the ARRIVE guidelines, and in accordance with national guidelines and regulations (GB 14925–2010) for reporting animal research as much as possible. The dandelion used in this experiment was purchased from Bozhou zhenshanyuan traditional Chinese medicine Sales Co., Ltd (Anhui Province, China). According to their instructions, the dandelion was cultivated in Bozhou in Northern Anhui Province of China, and the cultivation procedure was performed in compliance with the Convention on Biological Diversity and the Convention on the Trade and Endangered Species of Wild Fauna and Flora. The samples of dandelion are preserved in the Inner Mongolia Herbivorous Livestock Feed Engineering Technology Research Center, Hohhot, China.

### Animal, experimental design and diets

The study was carried out in compliance with the ARRIVE guidelines and national guidelines and regulations (GB 14925–2010). One-hundred and sixty 1-day-old AA male broiler chickens with similar birth weights and good physical condition were purchased from Inner Mongolia Yuean breeding Co., Ltd. The chickens were randomly divided into four dietary treatments: (1) CON (control, corn-soybean meal basal diet without additives, (2) LD (500 mg/kg dandelions of diet), (3) HD (1000 mg/kg dandelions of diet) and CTC (250 mg/kg chlortetracycline 20% premix of diet). Each group comprised five replicates with eight chickens per replicate. The experimental period was 42 days, which consisted of two phases: a starter phase (d 1 to 21) and a grower phase (d 22 to 42). The whole parts of dandelion were dried, grounded, and added to the basal diets. The polyphenols, flavonoids and polysaccharides contents of dandelion were 43.48, 35.00 and 193.93 mg/g, respectively. All basal diets were formulated to meet the nutrient recommendations of Feeding Standard of Chicken, China (NY/T 33–2004) (Chinese Ministry of Agriculture, 2004). Components of the diets, including crude protein (CP), ether extract (EE), crude fiber (CF), calcium, and phosphorus were measured according to the methods of the Association of the AOAC (2000). The concentrations of amino acid in diets were analyzed using HPLC (L-8900 AA Analyzer, Hitachi, Tokyo, Japan) as described previously [46]. The compositions and nutrient levels of the basal diets for starter and grower phases are presented in Table 8.

**Table 8:** Dietary composition and nutrient levels of basal diets

Items	Starter phase (d 1 to 21)	Grower phase (d 22 to 42)
Ingredient (%)		
Corn	52.50	58.80
Soybean meal	40.00	33.80
Soybean oil	3.00	3.00
Dicalcium phosphate	1.90	1.80
Limestone	1.08	1.22
Salt	0.37	0.37
Lysine	0.05	0.03
Methionine	0.19	0.07
Premix^1^	0.80	0.80
Choline	0.11	0.11
Total	100.00	100.00
Nutrient levels^2^		
Metabolic energy (MJ/kg)	12.42	12.62
Crude protein (%)	22.27	19.24
Ether extract (%)	4.69	5.45
Crude fiber (%)	3.50	3.26
Ca (%)	1.17	1.18
Total phosphorus (%)	0.82	0.77
Non-phytate phosphorus (%)	0.53	0.49
Lysine (%)	1.39	1.22
Methionine (%)	0.35	0.32
Threonine (%)	0.90	0.80
Tryptophan (%)	0.78	0.70

^1^The premix provided the following per kg of diet: VA, 9000 IU; VD_3_, 3000 IU; VE, 26 mg; VK_3_, 1.20 mg; VB_1_, 3.00 mg; VB_2_, 8.00 mg; VB_6_, 4.40 mg; VB_12_, 0.012 mg; nicotinic acid, 45 mg; folic acid, 0.75 mg; biotin, 0.20 mg; choline, 1100 mg; D-pantothenic acid, 15 mg; Fe, 100 mg; Cu, 10 mg; Zn, 108 mg; Mn, 120 mg; I 1.5 mg; Se, 0.35 mg

^2^Metabolic energy is a calculated value, while the others are measured values

Chickens had free access to feed and water throughout the trial period. The house temperature was set at 35℃ for 1 week, then gradually reduced by 2–3℃ per week to a 25℃ until the end of the trial. The lighting schedule was set as 24 h of lighting on during d 1–3, and 16 h of lighting:8 h of darkness up to d 14, then for natural light until d 42.

### Growth performance

Body weight (BW) of each bird and feed consumption of each replicate were measured at d 21 and d 42. The average daily gain (ADG), average daily feed intake (ADFI), and feed to gain ratio (F/G) were calculated.

### Sample collection

At d 21 and d 42, one bird was randomly selected from each replicate for sampling. After euthanizing by cervical dislocation, the gastrointestinal tract was removed. The ileal digesta was squeezed into a sterile tube, frozen in liquid nitrogen, and stored at -80℃ until further microbiota and SCFA analysis. The ileal tissues were washed with PBS buffer, frozen in liquid nitrogen and stored at -80℃ for gene expression and ELISA analysis.

### Gene expression analysis

Total RNA extraction from ileal tissue was performed using TRIzol reagent (Invitrogen, USA) according to the manufacturer’s instructions. The RNA integrity was determined by agarose gel electrophoresis, and the purity and the yield of the RNA was determined by NanoDrop ND-2000 spectrophotometer (Thermo Fisher Scientific). Total RNA (1 mg) was reversely transcribed to single-stranded cDNAs using PrimeScript™ RT reagent Kit with gDNA Eraser (Takara Bio Inc., Otsu, Japan). Real-time PCR was performed using the SYBR® Premix Ex TaqTM II (Takara Bio Inc., Otsu, Japan) in an ABI 7500 system (Applied Biosystems, Foster City, CA, USA). The reaction conditions were 95 °C for 30 s followed by 40 cycles of 95 °C for 5 s and 60 °C for 40 s. The specific sequences of primers for target genes (claudin, occludin-1, ZO-1 and mucin1) and endogenous reference gene (β-actin) are listed in Table 9. The relative mRNA expression of the target genes was calculated using the 2 ^−△△CT^ method [47]. Data for each target transcript were normalized to the control birds (1.0).

**Table 9:** Sequences of primers used in this study

Gene	Primer sequences (5’ → 3’)	Product size (bp)	Gene ID
Claudin	F:CCGCTGTCCTGAGCAGAGTT	161	424910
	R:TTTCCAGTGGCGATACCTAC		
Occludin-1	F:GGTTCCTCATCGTCATCCTG	149	396026
	R:TTCTTCACCCACTCCTCCAC		
ZO-1	F:CTAAGGGGAAGCCAACTGAT	215	415388
	R:ATTCTGAGGTGGAGGAGGGT		
Mucin1	F:ATATCCGTGCCGCTGGTT	239	426412
	R:GCCGGGCGTTGTTAATGT		

### Ileal immune factor analysis

The ileal tissue samples were homogenized in PBS, and the supernatants were obtained by centrifugation at 3500 r/min for 10 min at 4 °C. The concentrations of secretory immunoglobulin A (sIgA), interleukin (IL)-1, IL-2, IL-10, and tumor necrosis factor-α (TNF-α) in the supernatant were measured using commercial ELISA kits (Wuhan ColorfulGene Biological Technology Co., LTD). The protein concentration of supernatant was detected by the BCA protein assay kit (Thermo Fisher Scientific). All assay procedures were strictly performed according to the instructions.

### Ileal microbiota analysis

Ileal microbiota from the digesta samples were analyzed using bacterial 16S rRNA gene sequencing refereed to the previous study [10]. Briefly, whole genomic DNA was extracted from ileal digesta samples using the QIAamp DNA Stool Mini Kit (QIAGEN, Germany) according to the manufacturer’s protocols. The targets in the V4 region of the bacterial 16S rRNA gene was amplified using universal primers with a PCR system. Amplicon libraries for all samples were quantified by Qubit 2.0 Fluorometer (Thermo Fisher Scientific, Waltham, US) and sequenced on an NovaSeq6000 platform by Novogene Bioinformatics Technology Co., Ltd. (Beijing, China) to generate 2 × 250 bp paired-end reads.

All reads were processed and analyzed using the following procedures. The sequences were trimmed according to the sample barcode and primer sequences, and then were merged using FLASH V1.2.7. Chimeric sequences were removed using USEARCH. Then tags were clustered into the operational taxonomic unit (OTU) using UPARSE V7.0.1001, applying 97% sequence similarity thresholds. The representative sequences for each OTU were annotated using RDP classifier V2.2 and the Greengenes database V13.5. The alpha and beta diversity were analyzed by QIIME V1.9.1.

### SCFA analysis

The SCFA (acetic acid, propionic acid, and butyric acid) concentrations of ileal digesta were measured with liquid chromatography as described by Shivatare et al. [23]. In short, the analysis was carried out with a Waters Acquity UPLC BEH C8 column (100 mm × 2.1 mm, 1.7 μm) using 0.1% aqueous formic acid (A) and methanol (B) as the binary solvent system. The flow rate was set at 0.3 mL/min. Column was thermostatically controlled at 45℃. Injection volume was set to 4 μL.

### Statistical analysis

Experiment data were collated and preliminary processed using Excel software. Data were subjected to ANOVA using the MIXED procedure of SAS (SAS 9.2, SAS Institute Inc., Cary, NC). Mean and standard error of the mean (SEM) were reported, and means were separated by LSD multiple comparisons. Differences were considered significant at *P* < 0.05, and tendency when 0.05 ≤ *P* < 0.10.

## Data Availability

The datasets used and/or analyzed during the current study are available from SRA NCBI repository under the Bioproject accession number (PRJNA808831).

## Abbreviations

CH: Chinese herbs
CON: The control treament
LD: The low dose of dandelion treatment
HD: The high dose of dandelion treatment
CTC: The chlortetracyline treatment
ADG: Average daily gain
ADFI: Average daily feed intake
F/G: Feed to gain ratio
IL: Interleukin
sIgA: Secretory immunoglobulin A
TNF-α: Tumor necrosis factor-α
SCFA: Short chain fatty acids
ANONA: Analysis of variance
ZO-1: Zonula occludens-1
